# Abscess of the Antrum Maxillare

**Published:** 1846-12

**Authors:** J. R. Dillingham

**Affiliations:** Dental Surgeon. Fairhaven


***Abscess of the Antrum Maxillare.***—Dear Sir, I herewith transmit to you an account of a case of the disease of the *antrum maxillare,* to which my attention was called some time ago. The treatment of these diseases very properly comes under the care of the dentist, and he should be abundantly qualified to undertake their treatment; but nevertheless, it is often the case that he is entirely ignorant of the first cause of the disease, as the present case will testify.

The *antrum maxillare* is very subject to inflammation and suppuration, caused by disease of the neighboring parts. The natural mucus of these cavities accumulating, irritates and produces irritation for its own exit. The pain caused by the inflammation of the antrum is, in most cases, first taken for the toothache. Sometimes the eye as well as the nose is affected, extending to the frontal sinuses in the forehead. At first the symptoms are not sufficient to distinguish the disease. Time will disclose the true cause of the pain.

But to the case. Some five months since, a gentleman called on me, wishing me to examine his mouth, stating that he had for the last two months a violent pain in the upper jaw, extending at times to the forehead—also, that he had, within the two months, five upper teeth extracted on the side where the pain existed, by a dentist who assured him the cause was disease at the roots of the teeth. Still the pain continued, and on examination I found that the antrum was in a diseased state, so much so that with a common lancet I easily effected an opening inside of the lip. A large amount of matter was at once discharged, and the pain ceased. In a few days the matter again collected, and he had the same pain as before. Finding that I could not effect a cure by an opening in that place, I at once made an incision through the partition between the root of the alveolar process and the antrum, and then inserted a small tube of silver, which was kept there until the inflammation subsided, and an effectual cure was obtained. This was far the most preferable, for you are then sure of having an opening as long as wished for; and not only that, a better chance is thus obtained for the admission of the syringe, which should always be used in diseases of this kind. J. R. Dillingham, Dental Surgeon.

*Fairhaven, July,* 1846. [*Bost. Med. and Sur. Jour.*

